# Screening for epistatic selection signatures: A simulation study

**DOI:** 10.1038/s41598-019-38689-2

**Published:** 2019-01-31

**Authors:** S. Id-Lahoucine, A. Molina, A. Cánovas, J. Casellas

**Affiliations:** 1 Centre for Genetic Improvement of Livestock, Department of Animal Biosciences, University of Guelph, Guelph, N1G 2W1 ON Canada; 2 Departament de Ciència Animal i dels Aliments, Universitat Autònoma de Barcelona, 08193 Bellaterra, Spain; 3 Departamento de Genética, Universidad de Córdoba, 14071 Córdoba, Spain

## Abstract

Detecting combinations of alleles that diverged between subpopulations via selection signature statistics can contribute to decipher the phenomenon of epistasis. This research focused on the simulation of genomic data from subpopulations under divergent epistatic selection (ES). We used D’_IS_^2^ and F_ST_ statistics in pairs of loci to scan the whole-genome. The results showed the ability to identify loci under additive-by-additive ES (ES_aa_) by reporting large statistical departures between subpopulations with a high level of divergence, while it did not show the same advantage in the other types of ES. Despite this, limitations such as the difficulty to distinguish between the quasi-complete fixation of one locus by ES_aa_ from other events were observed. However, D’_IS_^2^ can detect loci under ES_aa_ by defining a minimum boundary for the minor allele frequency on a multiple subpopulation analysis where ES only takes place in one subset. Even so, the major limitation was distinguishing between ES and single-locus selection (SS); therefore, we can conclude that divergent locus can be also a result of ES. The test conditions with D-statistics of both Ohta (1982a, 1982b) and Black and Krafsur (1985) did not provide evidence to differentiate ES in our simulation framework of isolated subpopulations.

## Introduction

Genetic selection and demographic events have contributed to current genetic diversity. Under positive selection, the frequency of the favourable allele rises rapidly. Simultaneously, genetic diversity of neutral markers linked to the favourable allele is also affected, referred to as “hitch-hiking”^1^. Selection or hitch-hiking mapping approaches exploit this phenomenon by searching for genomic regions with reduced variability as signatures of selection^2–4^. This may contribute knowledge to the evolution and biology underlying a given phenotype, finding genomic regions controlling complex traits, and even identify candidate genes^5^.

Epistasis is the nonlinear interaction among loci, where the phenotype depends on a combined set of alleles at more than one locus. Under this phenomenon, the frequencies of favourable combinations of alleles increases in a population, and consequently stable linkage disequilibrium (LD) are expected^6^. Previously, Takahasi and Tajima^7^ studied the role of ES in the evolution of a coadapted haplotype within a population and Takahsi^8^ evaluated the effects of migration and isolation in a subdivided population. More recently, Behrouzi and Wit^9^ developed copula graphical models to detected ES in recombinant inbred lines. Here, we are approaching divergent epistatic selection across subpopulations exploiting selection signature statistics. In fact, in order to clarify the mechanisms responsible for LD, Ohta^6,10^ described the components of variance of LD (D-statistics). They accounted for within- and between-subpopulation effects in analogy with Wright’s^11^ F-statistics (see Supplementary Note online for more details). Several tests to discriminate among possible evolutionary forces shaping this variation using D-statistics were suggested by Ohta^6,10^ and Black and Krafsur^12^, such as ES and limited migration. Recently, Beissinger *et al*.^13^ reported that one of Ohta’s statistics^6,10^ may be capable of identifying pairs of loci that are jointly impacted by ES or single-locus selection (SS) in divergence subpopulations. Although it presented difficulties when trying to distinguish among both genetic mechanisms. The main objective of this research is to evaluate selection signature statistics to detect ES on simulated data sets in order to find a useful methodology to identify interactions between genes at the genome level.

## Methods

### Genome and population structure

This research focused on ES by simulation procedures in forward approach. Diploid individuals with two chromosomes were considered. Each chromosome was of 100 cM long with 2,000 biallelic markers uniformly distributed and ruled by a probability of mutation of 5·10^−4^, a plausible rate between a realistic probability and the density of markers assigned. Recombination events followed Kosambi’s function^14^ to reproduce LD. Each population was founded by 100 heterozygous individuals for all markers and evolved under random mating at a 1:1 sex ratio during 1,000 non-overlapping generations. This part of the simulation allowed us to generate a population of N_e_ = 100 with a biologically plausible genome. A minimum of allele frequency (MAF) of 0.25 were enforced for the specific regions where selection will be applied (first marker of each chromosome).

### Simulation selection scenarios

From generation 1001, two samples of N = 100 (or N = 500) were separated in order to start the divergence process of subpopulations. Three different divergent ES^15^ were considered: additive-by-additive (ES_aa_), additive-by-dominance (ES_ad_) and dominance-by-dominance (ES_dd_; Table 1). In order to contrast ES with single-locus selection, five different scenarios of SS were studied: additive (SS_a_) and dominance (SS_d_) where SS was applied in one SNP, additive and additive (SS_aa_), additive and dominance (SS_ad_) and dominance and dominance (SS_dd_) where SS was applied in two SNPs in an independent way. Advantageous and disadvantageous combinations of alleles created by additive and dominance interaction of genes (Table 1) were used to generate divergent subpopulations by simulating the opposite direction of selection between them. In this regard, specific genotypes were favoured in one subpopulation whereas being selected against them on the other subpopulation by changing the direction of selection; the positive (+1), neutral (0) or negative (−1) (Table 1).

**Table 1:** Different types of epistatic interaction and selection strategies simulated.

	ES_aa_			ES_ad_			ES_dd_		
	A1A1	A1A2	A2A2	A1A1	A1A2	A2A2	A1A1	A1A2	A2A2
B1B1	**1**	0	**−1**	0	**1**	0	0	0	0
B1B2	0	0	0	0	0	0	0	**1**	0
B2B2	**−1**	0	**1**	0	**−1**	0	0	0	0

ES: epistatic selection; aa: additive-by-additive; ad: additive-by-dominance; dd: dominance-by-dominance.

ES were parameterized such as the probability of favouring individuals to be selected according to the genotypes of two SNPs (located on two different chromosomes), while SS considered the selection on one unique SNP or two SNPs in an independent way. This probability was modeled by selection intensity (SI) and was set equal to (1) if the genotype of the individual corresponded to the genotype under positive selection, was set equal to (1–SI) when the genotype coincided with the neutral genotype and was set equal to (1–2·SI) when it matched the genotype under negative selection. Values of 0.05, 0.25 and 0.4 were tested for SI. Different numbers of generations (nG) under selection were generated, i.e., 5, 15 and 25 generations, and only the last generation contributed genomic data for further analyses. A total of 100 replicates were analysed per scenario.

### Statistical tests and implementation

For the analyses, two representative selection signature statistics were chosen. The conventional F_ST_^11^ is an example of a statistics based on allelic frequencies, and this quantifies the level of differentiation between populations^16^ by using components of variance of allele frequencies. On the other hand, D’_IS_^2^ of Ohta^6,10^ is a statistic based on haplotypes^13^, and is defined as the variance of the correlation of a pair of loci on the same gamete in a subpopulation relative to that of the average gamete of the population (see Supplementary Note online for Ohta’s statistics^6,10^). It is worthy to mentioned that F_ST_ is widely used to detect signature selection between breeds and D’_IS_^2^ was potentially suggested to be able to capture epistatic selection. For the analyses, ad hoc Fortran programs were developed based on the unbiased estimator of F_ST_ of Weir and Cockerham^16^ and the original formulation of D’_IS_^2^ of Ohta^6,10^. To scan the whole-genome we used sliding pairs of loci that combined two regions (2 nucleotide sites), each one from different chromosomes.

## Results and Discussion

### Effects of epistatic selection

The expected values of D’_IS_^2^ and F_ST_ are zero under null selection. Those statistics rose by the level of divergence between subpopulations (i.e., selection intensity and the number of generations since divergence). In this sense, high SI (≥0.4) and many generations (≥25) are mandatory to obtain divergent subpopulations and consequently to guarantee a statistical power for regions under selection. Loci with ES_aa_ provided high estimates (the top ~0.1% values) for D’_IS_^2^ (~0.97) and F_ST_ (~0.65) whereas moderate (~0.5; ES_ad_) and even lower estimates (~0.2; ES_dd_; Table 2) were shown by other ES models. In addition, including short-range LD by increase the length of pairs of loci (i.e., including additional adjacent SNP in each loci) showed also signal of selection but this decreased with the length in comparison to the two individual SNPs under ES (results not shown). This maximal departure generated by ES_aa_ was given by its ability to generate quasi-complete fixation of opposite alleles in one locus across subpopulations. In fact, different combinations of alleles are favoured within the same subpopulation by ES_aa_ (e.g., A1_B1 and A2_B2), but just one combination tended to spread within a subpopulation by chance (96% of times). Notice that the mating of parents with different alleles in one locus generated heterozygote individuals, which were discarded to be progenitors for the next generation with high probability and resulted more advantageous the propagation of one single type of homozygotes within a subpopulation. Indeed, high estimates were not expected when more than one combination of alleles resides in the same subpopulation. Specifically, the 4 replicates that kept play morphic under the ES_aa_ model, exhibited averages of 0.61 (±0.039) and 0.45 (±0.036) for D’_IS_^2^ and F_ST_, respectively.

**Table 2:** Average estimates of statistics (±s.d.) for loci under divergent selection across two subpopulations.

	SI	nG	D’_IS_^2^	F_ST_
ES_aa_	0.05	5	0.016 (±0.014)	0.020 (±0.023)
		15	0.054 (±0.061)	0.076 (±0.068)
		25	0.130 (±0.100)	0.171 (±0.116)
	0.4	5	0.255 (±0.098)	0.202 (±0.099)
		15	0.880 (±0.139)	0.609 (±0.076)
		25	0.974 (±0.078)	0.651 (±0.043)
ES_ad_	0.05	5	0.017 (±0.017)	0.023 (±0.029)
	0.4	25	0.500 (±0.016)	0.495 (±0.008)
ES_dd_	0.05	5	0.015 (±0.014)	0.019 (±0.023)
	0.4	25	0.183 (±0.067)	0.257 (±0.056)
SS_aa_	0.05	5	0.019 (±0.014)	0.027 (±0.026)
	0.4	25	0.975 (±0.030)	0.658 (±0.007)
SS_ad_	0.4	25	0.724 (±0.058)	0.576 (±0.021)
SS_dd_	0.4	25	0.367 (±0.063)	0.388 (±0.038)
SS_a_	0.05	5	0.020 (±0.018)	0.028 (±0.030)
	0.4	25	0.622 (±0.105)	0.535 (±0.034)
SS_d_	0.4	25	0.209 (±0.085)	0.291 (±0.069)

SI: selection intensity; nG: number of generations; ES: epistatic selection; SS: single-locus selection; a: additive; d: dominance.

### Genome-wide scan of epistatic selection

**Figure 1:**
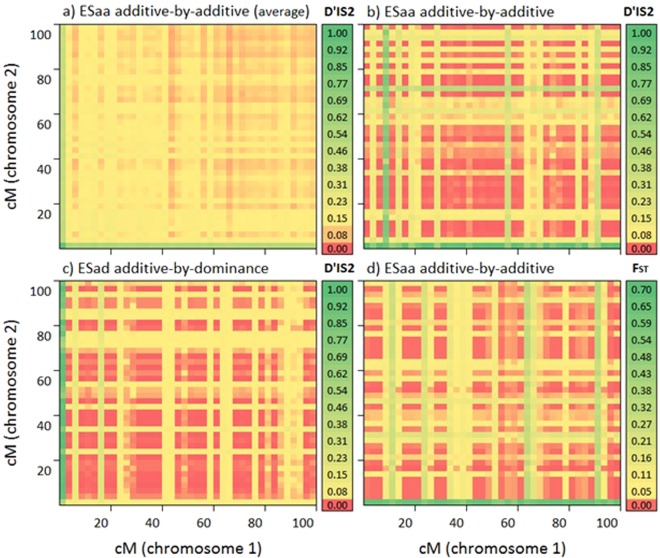
Genome-wide scan of two chromosomes by pairs of loci using estimates of D’_IS_^2^ (**a**–**c**) and F_ST_ (**d**) statistics for additive-by-additive (**a**,**b**,**d**) and additive-by-dominant epistatic selection (**c**). The subfigure (**a**) represents the average of 100 replicates. Regions under selection correspond to combinations of the first SNPs of both chromosomes (bottom-left of figures).

Four examples of genome-wide scans representing the estimates of statistics for each pair of loci composited by two SNPs of different chromosomes are shown in Fig. 1. Average results for D’_IS_^2^ showed high and remarkable estimates (~0.97; SI = 0.4 and nG = 25) when involving pairs of loci including both SNPs under ES_aa_ (Fig. 1a). Moderate values for D’_IS_^2^ (~0.4 to 0.6) were observed in pairs of loci including one SNP under selection and one unselected SNP on the alternative chromosome. Remaining pairs of loci that did not include SNPs under selection provided weaker values (≤0.2). The results (Fig. 1a) based on the average of the 100 replicas, showed an ideal situation where the two loci under ES were clearly differentiated with high and remarkable estimates of the statistics. However, when focusing in a more realistic situation (a single simulation), part of the effect of selection dragged across one of the chromosomes, specifically in the pairs of loci that included the divergent SNP under ES_aa_ with quasi fixed marker from the other chromosome (Fig. 1b). The same pattern was shown by ES_ad_, although maximum estimates of D’_IS_^2^ and F_ST_ may be obtained elsewhere and not concretely in both SNPs under ES_ad_ (Fig. 1c). In essence, we observed that any divergent locus across subpopulations combined with a fixed marker provided high estimates for D’_IS_^2^ and F_ST_.

This result highlighted the first difficulty of differentiating ES. As we discussed before, by chance, only one single combination of alleles arose within a subpopulation in most cases. This behaviour jointly with the parameterization of divergent ES_aa_ itself (Table 1) generated opposite alleles in one locus whereas the same alleles became fixed in the second locus in both divergent subpopulations (e.g., A1_B1/A2_B1). In this case, the difficulty resides in the differentiation between the fixation produced by ES_aa_ from other events. The use of a boundary for the MAF to discard markers with quasi-complete fixation across subpopulations may be an appealing solution but also can discard markers that had been fixed by ES. Within this context, a plausible scenario where ES can be differentiated could be in a multiple subpopulation analysis where ES takes place in one subset of them. Considering this, if one locus were led to fixation by ES, likely the SNP would become fixed only in subpopulations where ES_aa_ takes place. That may suppose a feasible behaviour for D’_IS_^2^ which kept displaying a high value when LD was presented only in some subpopulations (due to its calculation method based in summation of the variances).

On the other hand, it was clear that apparent selection signatures were observed in pairs of SNPs where no selection had been simulated; the same was reported by Vilas *et al*.^17^ in simulation data which can be explained by genetic drift. This kind of selection generated by genetic drift also showed patterns of selection across the whole chromosome. The markers that randomly diverged across subpopulations displayed high estimates of the statistics when combined with quasi fixed markers. However, we observed that the effect of genetic drift is minimized in large subpopulation size (N = 500). In principle, it is expected that the probability of a specific SNP to reach fixation at random must be low in small subpopulations. Within the same context, Rothammer *et al*.^18^ found a negative correlation between the number of detected signatures and N_e_ using real data from cattle breeds. In this context, in order to discard locus that had been diverged only by drift, large sample sizes would be required in the ideal scenario of multiple subpopulations.

### Interference of single-locus selection

Independent single-locus selection applied in one or two loci produced different patterns of the selection signature statistics. SS_a_ and SS_ad_ presented moderate-to-high estimates (~0.5 to 0.7), whereas SS_d_ and SS_dd_ showed lower-to moderate estimates (~0.2 to 0.35; Table 2). Part of the single replicates of SS_a_ also displayed a high estimate of the statistics, basically when the second locus became fixed by chance. In addition, when two loci were under SS_aa_, average estimates for D’_IS_^2^ and F_ST_ showed similar values obtained in loci under ES_aa_ model. At a genome-wide scan level, similar patterns to ES_aa_ were obtained in scenarios with loci under SS_a_, where high values were observed in most of the pairs of loci across one of the chromosomes combined with SNP under divergent selection. These patterns of selection were observed simultaneously in both chromosomes in the SS_aa_ scenarios. Nevertheless, the latter patterns were also produced in some regions where no selection had been simulated, thereby manifesting an apparent selection. This result added another difficulty of differentiating ES, related to differentiation between ES and SS, earlier noticed by Beissinger *et al*.^13^ for D’_IS_^2^. In fact, a simple additive selection may eventually lead to the formation of epistasis systems^7^. However, notice that statistical epistasis does not necessarily imply a functional epistasis.

### Test condition of the components of variance of linkage disequilibrium

Finally, the test condition containing D-statistics that Ohta^6,10^ and Black and Krafsur^12^ suggested to discriminate ES, did not provide evidence to differentiate ES in our simulation framework of isolated subpopulations. Whether the loci were under divergent ES or in the same direction across subpopulations, the results did not adjust with the latter tests to detect ES at a genome-wide level (results in the Supplementary Note). Under the same direction of selection across subpopulations, similar combinations were favoured, and in both subpopulations the same allele became fixed in most cases. In this case, D-statistics displayed low and extremely low estimates (close to 0) which do not have clear differences to contrast the test conditions of epistasis. Furthermore, when an allele has not yet been completely fixed within a subpopulation, the D-statistics fulfilled drift conditions instead of ES given the isolated subpopulations. On the other hand, for the test suggested by Black and Krafsur^12^, we noted that when ES takes place only in a subset of subpopulation the condition D_ST_^2^ < D_IS_^2^ for ES failed. By adding one unselected subpopulation in the analyses, the results of this work suggested that D_ST_^2^ was greater than D_IS_^2^, because it was expected that D_ST_^2^ increases (the averages over subpopulations change), while D_IS_^2^ continued close to 0 (it considers only the variance of LD of the own subpopulation). Similarly, the conditions of ES were fulfilled in some regions without any type of selection.

## Conclusion

Selection signature statistics explored in this study could identify additive-by-additive epistatic selection in divergent subpopulations with large statistical departures, whereas still unclear in other types of ES. Nevertheless, this method was unable to distinguish between the quasi-complete fixation produced in one locus by ES_aa_ from other events. D’_IS_^2^ could succeed in detection of ES_aa_ on multiple subpopulations analysis where it takes place in only one subset by defining a MAF to discard markers which reached fixation by other events. However, the discrimination between ES and SS remains to be the major limitation of this methodology; therefore, we can conclude that divergent loci may be a result of SS as well as ES. The test of both Ohta^6,10^ and Black and Krafsur^12^ did not provide evidence to differentiate ES in our simulation framework of isolated subpopulations (either under divergent selection as not).

## Supplementary information

Supplementary Note

Supplementary Note

## Data Availability

The datasets generated and analyzed during the current study are available from the corresponding author on reasonable request.
