# Supplementary material for: Screening for epistatic selection signatures: A simulation study
Source: Sci Rep. 2019 Jan 31;9:1026. doi: 10.1038/s41598-019-38689-2 (PMC6355851; doi:10.1038/s41598-019-38689-2)
Supplement: Supplementary file 2 — Supplementary Note [file 41598_2019_38689_MOESM2_ESM.pdf]

Screening for epistatic selection signatures: A simulation study

S. Id-Lahoucine[1,2,*], A. Molina[3], A. Cánovas[1] and J. Casellas[2]

[1]Centre for Genetic Improvement of Livestock, Department of Animal Biosciences, University of Guelph, Guelph, N1G 2W1 ON, Canada.

[2]Departament de Ciència Animal i dels Aliments, Universitat Autònoma de Barcelona, 08193 Bellaterra, Spain.

[3]Departamento de Genética, Universidad de Córdoba, 14071 Córdoba, Spain.

*Corresponding author: sidlahou@uoguelph.ca

Supplementary Note: Test condition of epistasis with components of variance of linkage disequilibrium (D-statistics)

The statistics developed by Ohta[1,2], considering two loci (A and B), are defined as: D_{IS}^2 is the expected variance of linkage disequilibrium within a subpopulation; D_{ST}^2 is the variance of the correlation of pair of loci (A and B) in one subpopulation relative to that of the total population; D_{IT}^2 is the total variance of disequilibrium, i.e., the expected variance of the correlation of pair of loci (A and B) of the same gamete in a subpopulation relative to that of the total population; $D'_{IS}{}^2$ is the variance of the correlation of a pair of loci (A and B) on the same gamete in a subpopulation relative to that of the average gamete of the population and lastly; $D'_{ST}{}^2$ is the variance of the disequilibrium of the total population. The D-statistics was computed as:

$$D_{IT}^2 = E\{\textstyle\sum_{i,j}(g_{ij,k} - \overline{x_i}\overline{y_j})^2\}, \; (1)$$

$$D_{IS}^2 = E\{\textstyle\sum_{i,j}(g_{ij,k} - x_{i,k}y_{j,k})^2\}, \; (2)$$

$$D_{ST}^2 = E\{\textstyle\sum_{i,j}(x_{i,k}y_{j,k} - \overline{x_i}\overline{y_j})^2\}, \; (3)$$

$$D'_{IS}{}^2 = E\{\textstyle\sum_{i,j}(g_{ij,k} - \overline{g_{ij}})^2\}, \; (4)$$

$$D'_{ST}{}^2 = E\{\textstyle\sum_{i,j}(\overline{g_{ij}} - \overline{x_i}\overline{y_j})^2\} \; (5)$$

where $x_{i,k}$ and $y_{j,k}$ were the frequencies of the ith and j^{th} alleles at loci A and B, respectively, in the kth subpopulation. The $g_{ij,k}$ was the frequency of gametes A_iB_j in the kth subpopulation and the $\overline{g}_{ij}$, $\overline{x}_i$ and $\overline{y}_j$ were averages over subpopulations.

According to the test of Ohta[1,2], when $D'^2_{IS} > D'^2_{ST}$ and $D^2_{ST} > D^2_{IS}$ were fulfilled, this suggests that genetic drift and limited migration is responsible for observed patterns of LD. Conversely, if epistatic natural selection is responsible for LD, it is expected that $D'^2_{IS} < D'^2_{ST}$ and $D^2_{ST} < D^2_{IS}$. The latter conditions had suggested, under the hypothesis, that the same combinations of alleles are being favoured consistently among subpopulations. Under a specific scenario where selection for particular pairs of loci occurs only in a subset of subpopulations, Black and Krafsur[3] proposed $D'^2_{IS} > D'^2_{ST}$ and $D^2_{ST} < D^2_{IS}$ condition to detect dispersive ES.

Table: Average estimates of D-statistics (±s.d.) for loci under ES_{aa} across subpopulations (SI=0.4; nG=25)

n subpop.	Dirc. selection	D'^2_{IS}	D'^2_{ST}	D^2_{ST}	D^2_{IS}
2	divergent direc.	0.974 (±0.078)	0.0001 (±0.0004)	0.969 (±0.103)	0.002 (±0.009)
	same direc.	0.192 (±0.388)	0.094 (±0.192)	0.286 (±0.579)	0.0003 (±0.002)
3	divergent direc.	1.063 (±0.070)	0.002 (±0.002)	1.059 (±0.079)	0.003 (±0.003)
	same direc.	0.509 (±0.316)	0.058 (±0.102)	0.562 (±0.413)	0.003 (±0.005)

Dirc.: direction, n subpop.: number of subpopulations (2 or 3 with one unselected subpopulation). Most replicates fulfilled the drift test condition and any with ES condition.

References

1. Ohta, T. Linkage disequilibrium due to random genetic drift in finite subdivided populations. *Proc. Natl. Acad. Sci. U. S. A.* **79**, 1940–4 (1982a).

2. Ohta, T. Linkage disequilibrium with the island model. *Genetics* **101**, 139-55 (1982b).

3. Black, I. V. W. C. & Krafsur, E. S. A FORTRAN program for the calculation and analysis of two-locus linkage disequilibrium coefficients. *Theor. Appl. Genet.* **70**, 491–96 (1985).
